# Revolutionizing Prostate Cancer Detection: The Role of Approved PSMA-PET Imaging Agents

**DOI:** 10.3390/ph18060906

**Published:** 2025-06-17

**Authors:** Ute Hennrich, Laurène Wagner, Harun Taş, Luciana Kovacs, Martina Benešová-Schäfer

**Affiliations:** 1 Service Unit for Radiopharmaceuticals and Preclinical Studies, German Cancer Research Center (DKFZ), Im Neuenheimer Feld 280, 69120 Heidelberg, Germany; 2 Research Group Translational Radiotheranostics, German Cancer Research Center (DKFZ), Im Neuenheimer Feld 280, 69120 Heidelberg, Germany; laurene.wagner@dkfz-heidelberg.de (L.W.); harun.tas@dkfz-heidelberg.de (H.T.); luciana.kovacsdossantos@dkfz-heidelberg.de (L.K.); m.benesova@dkfz-heidelberg.de (M.B.-S.)

**Keywords:** prostate cancer, PSMA, PET diagnostics, fluorine-18, gallium-68, FDA approval, EMA approval, Locametz^®^, Illuccix^®^, Gozellix^TM^, Pylarify^®^, Pylclari^®^, Radelumin^®^, Posluma^®^

## Abstract

Locametz^®^/Illuccix^®^/Gozellix^TM^ (Novartis AG (Basel, Switzerland) and Telix Pharmaceuticals, Ltd. (Melbourne, Australia), all three [^68^Ga]Ga-PSMA-11), Pylarify^®^/Pylclari^®^ (Progenics Pharmaceuticals, Inc. (New York, USA) and Curium PET France SA (Paris, France), both [^18^F]DCFPyL), Radelumin^®^ (ABX GmbH (Radeberg, Germany), [^18^F]PSMA-1007), and Posluma^®^ (Blue Earth Diagnostics, Ltd. (Oxford, UK), [^18^F]rhPSMA-7.3) are four approved PSMA-PET imaging agents that have significantly advanced the diagnosis and management of prostate cancer. These agents offer a new level of precision and accuracy, enabling clinicians to detect prostate cancer with enhanced sensitivity. As a result, they play a critical role in improving detection, staging, and management, ultimately enhancing clinical outcomes for patients. Their use in routine clinical practice is expected to increase diagnostic precision and provide clearer pathways for personalized therapy. This review offers a comprehensive chemical, pharmaceutical, and medicinal overview, discusses comparative studies, and highlights additional highly relevant candidates for prostate cancer detection.

## 1. Introduction

Prostate cancer (PCa) remains a significant public health challenge globally. It is the second most common cancer diagnosed in men worldwide (14.2%), behind lung cancer (15.3%) [1], and it is the most frequently diagnosed cancer in 118 of the world’s 185 countries. Estimates indicate that the numbers of new cases will increase annually from 1.4 million in 2020 to 2.9 million in 2040 [2]. Although PCa is highly prevalent, it ranks fifth among the leading causes of cancer-related deaths (7.3%) worldwide. The impact of PCa varies among different regions. It is the leading cause of cancer-related deaths in 52 countries, with some of the highest mortality rates in the Caribbean and sub-Saharan Africa, highlighting significant disparities in early detection, prevalence of risk factors and treatment availability [1]. Notably, Black men have the highest cancer mortality rates, which are two-to-four times higher than those of other ethnic groups [3,4]. PCa is a complex and heterogeneous disease, characterized by multiple genomic alterations [5,6,7]. In its early stages, it is often asymptomatic, with symptoms typically appearing only when the disease is already advanced, thus limiting treatment options and decreasing the chances of survival. This highlights the importance of early and accurate detection for a more favorable prognosis.

PCa is usually diagnosed by a combination of different methods, digital rectal examination (DRE), a blood test to measure prostate-specific antigen (PSA) levels, and transrectal ultrasound-guided (TRUS) prostate biopsy, which provides the final confirmation. The DRE was the only screening test for PCa until the discovery of PSA [8,9]. However, its performance for the initial detection of PCa is limited and there is a high degree of interobserver variability [8,10]. PSA is a serine protease, discovered in 1971, and its physiological function is to liquefy seminal fluid. High serum PSA levels are associated with PCa, and PSA is widely recognized as a biomarker for the early detection, staging and monitoring of response to treatment. Serum PSA measurement is specific for the prostate but is not specific for PCa, as benign prostate diseases, age, body mass index, and race also significantly influence blood PSA levels [11]. The blood PSA levels cannot be used as a screening test for PCa alone and TRUS prostate biopsy has been the standard diagnostic test to confirm PCa [7,12]. TRUS is widely used due to its accessibility and cost; however, the diagnostic accuracy is highly dependent on the operator and random needle placement relative to the tumor, leading to the risk of sampling error and high rate of false negative results (15 to 46%) [7]. Magnetic resonance imaging (MRI) and MRI-fusion-guided biopsy has been used to improve diagnostic accuracy in PCa with positive results; however, studies have shown that MRI fails in the detection of all cancer sites and, in some cases, underestimates the tumor size, leading to inadequate treatment [12,13]. The lack of accurate diagnostic methods for PCa has resulted in unnecessary biopsies and overdiagnosis, highlighting the urgent need for alternative diagnostic modalities with greater specificity and sensitivity.

The use of positron emission tomography (PET) with prostate-specific membrane antigen (PSMA) imaging is revolutionizing the management of PCa, making diagnosis more accurate and treatment more effective [14,15]. PSMA is a transmembrane glycoprotein, first identified in the late 1980s by Horoszewicz and colleagues [16]. PSMA—also called glutamate carboxypeptidase II (GCPII), *N*-acetyl-aspartylglutamate (NAAG) peptidase or *N*-acetyl-l-aspartyl-l-glutamate peptidase I (NAALADase I) and folate hydrolase (FOLH1)—is involved in the enzymatic process of glutamate release, folate hydrolase activity and several enzymatic functions [17]. It is primarily expressed in the prostate, with lower levels found in the salivary gland, brain, small intestine and kidney, and is implicated in PCa and neurological disorders. PSMA is overexpressed in PCa cells, in the primary tumor and in metastases, as well as in the angiogenic vasculature of several solid tumors, including lung, breast, kidney, bladder, ovarian and colon cancers, as well as glioblastomas [18,19]. In the last two decades, PSMA has become a valuable target for the diagnosis and therapy of PCa due to its high expression in malignant prostate cells. Its expression levels correlate with tumor progression, cancer aggressiveness and poor prognosis, making it a biomarker for disease assessment and therapy.

PSMA-targeted imaging using PET allows for the simultaneous detection of primary tumors and metastases with higher accuracy than conventional methods. PET techniques offer high sensitivity and specificity, giving a good overview of the disease and leading to a more accurate diagnosis. These advantages play a critical role in early diagnosis, therapy monitoring, and understanding disease mechanisms, transforming PCa diagnostics. Currently, there are four PSMA-targeting radiotracers approved for the PET imaging of PSMA-positive PCa. The FDA approved three [^68^Ga]Ga-PSMA-11 kits: Illuccix^®^ in December 2021, Locametz^®^ in March 2022, and Gozellix^TM^ in March 2025. Until now, in Europe, only Locametz^®^ has been commercially available (approved by the EMA in December 2022), but the EMA issued a positive opinion for Illuccix^®^ in January 2025 as well. [^18^F]DCFPyL received FDA approval in May 2021 (Pylarify^®^), and in July 2023 in Europe (Pylclari^®^). [^18^F]rhPSMA-7.3 (Posluma^®^) was approved by the FDA in May 2023. Finally, [^18^F]PSMA-1007 (Radelumin^®^) was initially approved in France in December 2022, with subsequent approvals in several other European countries. In general, PET imaging targeting PSMA is indicated to assess the presence of metastases in patients with PCa before treatment begins, to evaluate PCa recurrence in patients with high PSA levels after treatment and to determine PSMA expression in cancer cells to guide eligibility for PSMA-targeted therapy. The approval of these radiopharmaceuticals for clinical use represents a significant advance in the diagnosis, treatment and management of PCa, enabling earlier detection of metastatic and recurrent disease. PSMA-targeted PET imaging has the potential to greatly influence clinical decisions and optimize treatment planning for patients. In this review, we discuss the four radiotracers approved for clinical use, highlighting the chemical characteristics, radionuclides, clinical applications and future prospects.

## 2. Chemical Overview

### 2.1. Names, Chemical Structures and Properties

Before being introduced as commercially available PET radiotracers with trade names, all PSMA inhibitors were initially referred to by working names, many of which are still widely used. Table 1 summarizes these various names, along with their respective manufacturers.

The IUPAC names of the radiotracers are as follows:

- **[^18^F]PSMA-1007**: (3S,10S,14S)-1-[4-[[(2S)-4-carboxy-2-[(2S)-4-carboxy-2-(6-[^18^F]fluoropyridin-3-amido)butanamido]butanamido]methyl]phenyl]-3-[(naphthalen-2-yl)methyl]-1,4,12-trioxo-2,5,11,13-tetraazahexadecane-10,14,16-tricarboxylic acid
- **[^68^Ga]Ga-PSMA-11**: [^68^Ga]gallium (3S,7S)-22-[3-[[[2-[[[5-(2-carboxyethyl)-2-hydroxyphenyl]-methyl](carboxymethyl)amino]ethyl](carboxymethyl)amino]-methyl]-4-hydroxyphenyl]-5,13,20-trioxo-4,6,12,19-tetraazadocosane-1,3,7-tricarboxylic acid
- **[^18^F]DCFPyL**: 2-(3-{1-carboxy-5-[(6-[^18^F]fluoro-pyridine-3-carbonyl)amino]-pentyl} [18]ureido)-pentanedioic acid
- **[^18^F]rhPSMA-7.3**: gallate(6-), [(4S,8S,13R,27R,30R,35S)-35-[4,10-bis[(carboxy-kO)me-thyl]-7-(carboxymethyl)-1,4,7,10-tetraazacyclododec-1-yl-kN^1^,kN^4^,kN^7^,kN^10^]-30-[[[4-[bis(1,1dimethylethyl)fluoro-^18^F-silyl]benzoyl]amino]methyl]-1,36-dihydroxy-1,6,11,18,21,29,32,36-octaoxo-5,7,12,17,22,28,31-heptaazahexatriacontane-4,8,13,27-tetracarboxylato(9-)]-, hydrogen (1:6)

**Figure 1:**
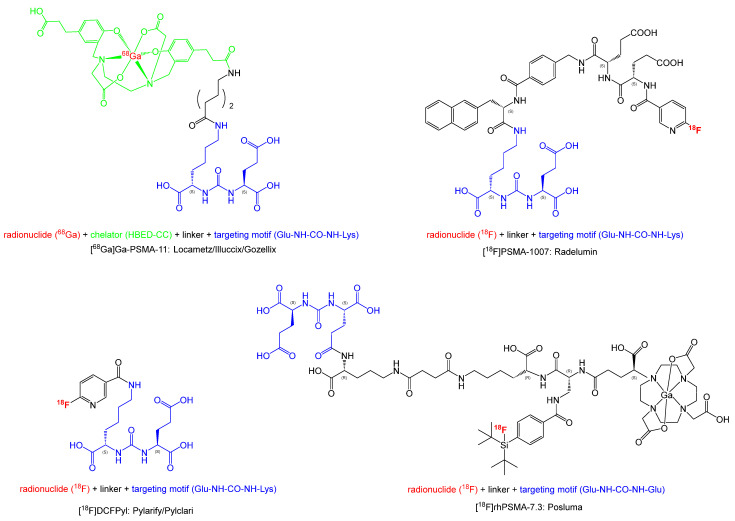
Chemical structures of approved PSMA-targeting PET radiotracers.

Figure 1 illustrates the chemical structures of the compounds. All four PET ligands are based on a urea motif which plays an important role in binding to PSMA. Although the radiotracers [^68^Ga]Ga-PSMA-11, [^18^F]DCFPyL, and [^18^F]PSMA-1007 share the Glu-NH-CO-NH-Lys binding motif to which, via different linkers, the radiolabeled moiety is attached, the chemical structure of [^18^F]rhPSMA-7.3 differs significantly. While the difference in the binding motif (Glu-NH-CO-NH-Glu) is slight, the rest of the molecule incorporates a chelator (DOTA-GA: 2-(4,7,10-tris(carboxymethyl)-1,4,7,10-tetraazacyclo-dodecan-1-yl)pentanedioic acid), as well as the silicon–fluoride acceptor (SiFA) moiety, offering two possibilities for radiolabeling: complexation of a radiometal such as gallium-68 (^68^Ga) or lutetium-177 (^177^Lu) with the DOTA-GA chelator or by the isotopic exchange reaction of fluorine-18 (^18^F) against fluorine-19 (^19^F) in the SiFA moiety [20]. This allows, in particular, for combining a diagnostic radiotracer and a therapeutic radiopharmaceutical in a single molecule, making it, theoretically, an ideal theranostic compound. For therapeutic applications, the radionuclide ^177^Lu is paired with non-radioactive ^19^F in the SiFA moiety, while for diagnostic purposes, ^18^F is combined with non-radioactive ^175/176^Lu in the chelating moiety. The combination of two radiolabeling possibilities tremendously enhances the complexity of the molecule, as well as its molecular weight (1537.3 g/mol vs. 441.4 g/mol for [^18^F]DCFPyL).

Looking at the radionuclide, three of the four PET radiotracers discussed are labeled with ^18^F and one with ^68^Ga. For ^68^Ga labeling, a chelator is needed, which, in the case of PSMA-11, is known as HBED-CC (*N*,*N*′-bis [2-hydroxy-5-(carboxyethyl)benzyl]-ethylenediamine-*N*,*N*′-diacetic acid). This acts as a hexadentate chelator with octahedral geometry and coordinates the radiometal to two nitrogen atoms, two hydroxy groups and two carboxylic groups [21,22]. Depending on the reaction conditions, such as temperature, time, and pH of the reaction solution, up to three different diastereomers of [^68^Ga]Ga-PSMA-11 can be formed. Since preclinical studies have shown that the diastereomers do not bind differently to the PSMA receptor, the PET image should not be influenced by them [22].

Looking at the ^18^F-labeled radiotracers, [^18^F]PSMA-1007 and [^18^F]DCFPyL present many similarities: in both molecules, the ^18^F label is incorporated into a nicotinic acid moiety. Both radiotracers can be synthesized by the direct fluorination of an unprotected trimethylammonium labeling precursor, where a trimethylammonium-leaving group is substituted by ^18^F [23,24]. For [^18^F]rhPSMA-7.3, radiolabeling occurs in a completely different way by an isotopic exchange reaction where the natural ^19^F is replaced by the radioactive ^18^F in the SiFA group. This labeling approach enables fluorinations under very mild reaction conditions (e.g., room temperature), thus allowing for access to molecules that may not be stable under the harsher conditions required for classical fluorinations [25]. Furthermore, there is no need to separate the labeling precursor from the product, since it is the same molecule. This lowers the specific activity of the tracer solution and might have an effect on tracer uptake. In the case of [^18^F]rhPSMA-7, there are four possible isomers which show partially different behavior in vitro and in vivo. Due to high tumor accumulation and low binding in blood, liver and kidneys, [^18^F]rhPSMA-7.3 has been shown to be the preferred isomer [26].

### 2.2. Radionuclides

As radionuclides, ^68^Ga or ^18^F are employed for the approved PSMA radiotracers. It is not possible to state categorically which PET radionuclide is more suitable, as both have distinct advantages and limitations. When analyzing the physical characteristics of both radionuclides, different parameters can be considered, which impact the image quality [27]. Although both radionuclides decay primarily by positron emission, the positron yield of ^68^Ga is only 89.1% (decaying to the stable isotope zinc-68 (^68^Zn)) compared to 96.8% for ^18^F (decaying to stable oxygen-18 (^18^O)), resulting in a lower detection sensitivity of ^68^Ga. This can be counteracted by higher initial activities or longer scan times. Furthermore, the maximum and average β^+^ energy of both nuclides differ: the maximum β^+^ energy for ^68^Ga is 1899.1 keV vs. 633.5 keV for ^18^F, and the average β^+^ energy is 836 keV vs. 249.3 keV. These energies directly impact the spatial resolution resulting from the mean positron range in soft tissue which is 1.05 mm for ^68^Ga compared to 0.27 mm for ^18^F. Considering these parameters, ^18^F is the better radionuclide.

Regarding the half-lives of both radionuclides, 67.7 min for ^68^Ga and 109.7 min for ^18^F, the considerably shorter half-life of ^68^Ga has several implications. The synthesis and quality control processes must be performed more rapidly, as the tracer decays continuously during this time, thereby reducing the amount of injectable radiotracer available. As a rule of thumb, the time until the release of the radiotracer should not exceed one half-life. Usually, the synthesis of ^68^Ga-labeled radiotracers is fast, consisting of the complexation of the radiometal, rarely removal of protecting groups, and most of the time, purification can be accomplished by solid phase extraction. For classical radiofluorinations, the synthesis time, including steps such as drying of the [^18^F]fluoride, labeling reaction, deprotection and purification, for which frequently high-performance liquid chromatography (HPLC) is necessary, is often longer. Therefore, considering this, there is no major advantage for one or the other radionuclide. However, when it comes to distributing the radiotracer to external hospitals instead of using it exclusively in-house, this is typically not feasible with ^68^Ga-labeled tracers produced via a ^68^Ge/^68^Ga generator, due to the short half-life and limited production capacity. Furthermore, using one batch of radiotracer for more than 1–2 patients is not possible with generator-produced ^68^Ga-labeled radiotracers, in contrast to ^18^F-labeled radiotracers, where multiple patients can be imaged with one batch. In recent years, the production of ^68^Ga with a cyclotron has been under investigation, delivering promising results. The production of ^68^Ga with a cyclotron can either be carried out by irradiation of a solid or liquid target containing enriched ^68^Zn. For the solid target, up to 192 GBq ^68^Ga can be obtained at the end of purification, as well as 72 GBq at end of synthesis (EOS) and for the liquid target up to 3 GBq (EOS) [28]. Especially regarding the activity amount achievable by solid target, a distribution of the final radiotracer is feasible. That the production of ^68^Ga by cyclotron can be an important route is also demonstrated by the fact that already in 2021 a monograph “gallium (^68^Ga) chloride (accelerator-produced) solution for radiolabeling” (01/2021:3109) was released in the European Pharmacopoeia [29]. In comparison, depending on the beam energy and beam current, up to 1110 GBq of [^18^F]fluoride (18 MeV proton beam @300 µA beam current) can be produced by irradiation of enriched H_2_^18^O [30]. Most of the time, these high amounts of radioactivity are not needed, and by adjusting beam parameters, like beam time or current, a suitable amount can be produced. When using high amounts of ^18^F, the problem of radiolysis may arise, hampering the stability of the radiotracer [31].

Considering all these facts, ^18^F seems to be the best choice as a radionuclide. But there are still cases when ^68^Ga is superior: one case is the possibility that the chosen biomolecule might not be amenable to radiofluorination. Especially considering the relatively harsh reaction conditions required (e.g., high temperatures), the molecule might not be stable and also the introduction of fluorine via nucleophilic or electrophilic substitution into the molecule might not be possible. With the introduction of modern techniques like labeling by isotopic exchange in the SiFA moiety [25] or the introduction of fluorine in the form of an aluminium–[^18^F]fluoride–chelator complex [32], this limitation might be overcome in the future. But at least when looking at the [Al^18^F]^2+^ complexes, there are still a lot of optimizations to be carried out, especially regarding the automation of the synthesis, which is essential for GMP compliance of the production of tracers for human use.

The most significant advantage of ⁶⁸Ga lies in the availability of various authorized ^68^Ge/^68^Ga generators on the market which can be used for the synthesis of ^68^Ga-labeled radiotracers for human use. This is a tremendous benefit for all radiopharmacies without a cyclotron on-site. Since, at least in Europe, the radiolabeling precursor [^18^F]fluoride for radiolabeling counts as a pharmaceutical and not a starting material, it is not allowed to purchase it commercially from another cyclotron facility, as long as they do not have marketing authorization [33]. Although there are initiatives for a change in the European directive, it is not clear if and when this change will happen. As of now, e.g., in Germany, when applying for a manufacturing authorization for a ^18^F-labeled radiotracer, the [^18^F]fluoride must be produced in-house or purchased from a facility holding a marketing authorization, which so far does not exist. And since authorized ^68^Ge/^68^Ga generators are commercially available, this problem does not apply to ^68^Ga. On the generator column, the bound parent nuclide ^68^Ge (half-life 271 d) continuously decays to its daughter nuclide ^68^Ga via electron capture. The daughter can be eluted multiple times per day with diluted hydrochloric acid and used in the labeling reactions. Even though authorized generators are quite expensive, they can be used for up to one year, if the decline in radioactivity to less than half is acceptable. As long as the generator is used in compliance with the supplier’s instructions, there is a guarantee that the breakthrough of the parent nuclide ^68^Ge, which would be critical, will not occur within a year. Therefore, for facilities without a cyclotron, the use of ^68^Ga-labeled tracers is very beneficial and may represent the only option for GMP-compliant production of radiotracers for human use.

### 2.3. GMP-Compliant Synthesis

In contrast to radiotracer production for research purposes, the production of radiotracers for human use has to fulfill complete GMP compliance. An important aspect is the appropriate environment, which requires dedicated clean rooms for the complex production of the tracers: all production steps have to be conducted in a class C clean room environment and sterile filtration of the final product, as well as its portioning even in a class A environment. This can be a major problem for smaller facilities, when they do not have appropriate clean rooms. Another part of GMP-compliant production is the automation of the reaction and purification steps. This can be accomplished in automated synthesis units (ASUs) where all steps are conducted with a computer program from outside the hot cell. No manual interference is allowed. There are a lot of different ASUs from various suppliers commercially available. In order to rule out cross contamination, nowadays mostly cassette-based ASUs are used, facilitating the production of more than one tracer on one unit. For many routinely used PET radiotracers, these disposable cassettes are available either by the vendor of the ASU or by special suppliers. All four approved radiotracers can be produced in ASUs and several optimizations have been performed by various groups.

As described above, there are many similarities between [^18^F]DCFPyL and [^18^F]PSMA-1007, which also results in similar synthetic routes for their radiolabeling. For both molecules, there are two possible radiolabeling approaches. One of them is the use of the secondary labeling precursor 6-[^18^F]fluoronicotinic acid tetrafluorophenyl ester ([^18^F]F-Py-TFP) [23,34]. In a first step, this labeling precursor has to be produced and purified. Then, it can be conjugated to the molecule of interest under relatively mild conditions, followed, if necessary, by a deprotection step and further purification of the final product by HPLC. This complex sequence of reactions results in long production times (128 min for [^18^F]DCFPyL [34] and 80 min for [^18^F]PSMA-1007 [23]) and can be difficult to implement in an ASU. Furthermore, optimization concerning the synthesis of [^18^F]F-Py-TFP still requires an overall production time for [^18^F]DCFPyL of 56 min [35]. Therefore, whenever possible, a synthetic route using direct radiofluorination of a suitable labeling precursor is preferable. In the case of both radiotracers, this approach is feasible using an unprotected trimethylammonium precursor which has been implemented and optimized on various ASUs (see literature examples for [^18^F]DCFPyL [24,34,36] and [^18^F]PSMA-1007 [23,37,38,39]). By enabling purification of the final product using solid phase extraction (SPE) and avoiding time-consuming HPLC purification, total production times can be reduced to as short as 21 min for [^18^F]DCFPyL [24] and 40 min for [^18^F]PSMA-1007 [38]. These automated production processes can be easily adopted in suitably equipped radiopharmacies and demonstrate a very high reliability for routine productions.

In contrast to these two ^18^F-labeled radiotracers, the labeling strategy of [^18^F]rhPSMA-7.3 differs significantly since the radiofluorine is incorporated into the SiFA moiety by an isotopic exchange reaction. Unlike for the other radiotracers, not a lot of optimizations and automated synthesis strategies have been reported in the literature, only by the group of Wester et al. [40]. These exchange reactions can be conducted at room temperature and azeotropic drying of the [^18^F]fluoride, as required for classical radiofluorinations to obtain a reactive fluoride complex, can be omitted. The separation of [^18^F]fluoride from irradiated target water on an SPE cartridge, washing with anhydrous acetonitrile and elution with a kryptofix/potassium hydroxide solution are sufficient. Therefore, a total reaction time of 16 min has been reported for the production of [^18^F]rhPSMA-7.3. However, this type of reaction can present some pitfalls, as an update on SiFA reactions describes that the elution cocktail of kryptofix/potassium carbonate has a very limited stability which might lead to problems in the production [41]. Furthermore, when taking a look at the achievable specific activities of SiFA-based compounds, they are significantly lower than for ^18^F-labeled radiotracers from conventional radiofluorinations. This results from the fact, that in SiFA reactions the labeling precursor is the same compound as the labeled tracer, with the only difference being the incorporation of non-radioactive ^1^⁹F. When comparing the amount of carrier which results in the specific activity of the tracer, it is ≤3.42 µg/GBq for Posluma^®^ (≤20 µg/mL in ≤5846 MBq/mL) [42]) vs. ≤0.27 µg/GBq for Pylarify^®^ [43,44]. This may have an impact on the uptake of the tracers and the image interpretation.

For the production of [^68^Ga]Ga-PSMA-11, there are two different possibilities: production in an ASU similar to those described for ^18^F-radiotracers or the use of authorized sterile cold kits, such as those that have been used for radiolabeling with ^99m^Tc for many years. When producing [^68^Ga]Ga-PSMA-11 in an automated synthesis, all rules for full GMP compliance have to be followed as explained above. This is not necessary for a preparation with sterile kits and an authorized sterile ^68^Ge/^68^Ga-generator. Since the synthesis takes place in a closed sterile vial, it only has to be guaranteed that the end product is sterile by preparation in a sterile environment like a laminar flow safety cabinet; a clean room is not mandatory. In typical automated synthesis, the elution and pre-purification of the ^68^Ga-eluate are followed by reaction with the labeling precursor PSMA-11 at elevated temperatures (e.g., 85 °C) and SPE purification, dilution and sterile filtration [45]. This results in production times of as short as 15 min and an RCY of 85.35 ± 5.78%. For this production route, every local radiopharmacy typically has to apply for some type of authorization with the local authorities since the decentralized production and distribution of the radiotracer are not feasible due to the short half-life of ^68^Ga. In order to simplify the entire labeling procedure and make it accessible to a larger number of locations, the production of [^68^Ga]Ga-PSMA-11 by use of sterile cold kits has been developed, and currently, there are three different approved kits on the market: Illuccix^®^/Gozellix^TM^ (both Telix Pharmaceuticals, Inc., Melbourne, Australia) and Locametz^®^ (Novartis AG, Basel, Switzerland). Illuccix^®^ has been the first kit to be approved by the FDA in 2021 [46] as well as in Canada and Australia, and recently, the EMA has given a positive approval statement in 2025 paving the path for use in Europe [47]. In March 2025, Gozellix^TM^, an improved version offering a longer shelf life due to the addition of ascorbic acid as a stabilizer, has also received approval by the FDA [48]. Locametz^®^ has been approved by the FDA and EMA in 2022 [49,50]. While the Locametz^®^ kit consists of one sterile vial including everything needed for a successful production, the Illuccix^®^ and Gozellix^TM^ kits have three vials (one containing reaction buffer, one PSMA-11 precursor, and one for elution of the generator) or two vials (reaction buffer, PSMA-11 precursor and an ampule with ascorbic acid), respectively, making their synthesis a bit more complicated. The production of [^68^Ga]Ga-PSMA-11 is described in the respective prescribing information of the kit [46,48,49,50], and is extremely easy to accomplish since it more or less consists of the elution of the generator, reaction at room temperature for 5 min and quality control of the end product. A very nice comparison of the Illuccix^®^ and Locametz^®^ kits, as well as optimizations and possible pitfalls of the labeling procedures, has been published recently [51]. Since all eluted radioactivity is available in the end product, the RCY is only diminished by the radioactive decay during synthesis and quality control. The required radiochemical purity of >95% is easily achieved. Even though the cold kits are quite expensive with costs well above EUR 1000 per kit, the cold kit production of [^68^Ga]Ga-PSMA-11 is by far the easiest and most accessible way to get a diagnostic PSMA radiotracer, especially since no manufacturing authorization is necessary but a notification for compassionate use at the local authorities is sufficient.

In contrast to ^68^Ga-labeled radiotracers, which must be produced on-site or nearby, ^18^F-labeled radiotracers can be shipped over considerable distances due to the longer half-life of ^18^F and the ability to produce ^18^F-labeled radiotracers with much higher activities. Therefore, shipping all three ^18^F-labeled PSMA tracers to other sites is entirely feasible, and all of the companies involved have multiple production sites to facilitate widespread distribution. While [^18^F]DCFPyL is available in large parts of the US [52] as well as Europe [53], [^18^F]rhPSMA-7.3 is distributed only in the US [54] and [^18^F]PSMA-1007 only in Europe [55].

### 2.4. End-Product Specifications and Quality Control

For patient safety, it is mandatory to check the quality of each radiotracer batch before application. The amount and type of analytical tests required may differ depending on local authorities as well as the compound and its synthesis route. All analytical methods must be validated according to GMP compliance. In order to pass quality control and be released for patient application, every radiotracer batch has to fulfill certain acceptance criteria (e.g., (radio)chemical purity, pH value, residual solvents and endotoxin content) which are defined in European Pharmacopeia monographs for [^68^Ga]Ga-PSMA-11 and [^18^F]PSMA-1007 [56], as well as adequate analytical methods. Until today, for [^18^F]DCFPyL and [^18^F]rhPSMA-7.3, no monographs exist but suitable acceptance criteria can be defined in analogy to other ^18^F-labeled radiotracers, as exemplified in the literature [34,40]. The most important parameter is the radiochemical purity of the tracer which can be determined by radio-HPLC and radio-TLC/iTLC. Using radio-HPLC, the RCP for [^68^Ga]Ga-PSMA-11 has to be ≥95%, for [^18^F]PSMA-1007 ≥ 91%. Generally, impurities like non-reacted ^68^Ga or ^18^F can be determined better with radio-TLC/iTLC than with radio-HPLC and they should not exceed 3% (^68^Ga) and 5% (^18^F), respectively. In addition, the chemical purity of the tracer is of utmost importance and acceptance criteria are specified in the respective monographs and in the literature. Due to the half-life, not all required analytical tests can be performed before release of the batch, e.g., sterility tests and tests on radionuclidic purity.

For [^68^Ga]Ga-PSMA-11 produced by authorized cold kits, only limited quality control has to be performed for each batch, as described in the label [46,48,49,50]. For radiochemical purity, radio-iTLC is sufficient and the chemical purity does not have to be determined at all. The only other parameters to be analyzed are the appearance (clear, without particles) and pH value (3.2–6.5 (Locametz^®^) and 4–5 (Illuccix^®^/Gozellix^TM^)). These tests confirm the quality of each batch and can be performed without the need for a more sophisticated quality control lab.

For ^18^F-labeled radiotracers, which are produced through much more complex reactions involving a greater number of chemicals, such limited quality control would not be sufficient. Besides tests like appearance, pH value and endotoxin content, a radio-HPLC analysis confirming radiochemical and chemical purity, as well as identity, is essential. When looking at the documentation of the commercially available ^18^F-labeled radiotracers, not a lot of information about their specifications is given. However, all release specifications have been approved by the respective authorities and must be met for each batch. Parameters which are publicly known are summarized in Table 2:

After quality control, which usually takes up to 30 min, the radiotracer batch can be released for patient application. When receiving a released batch of an authorized radiotracer, only the appearance and radioactivity amount have to be checked.

## 3. Medicinal and Pharmaceutical Overview

### 3.1. Clinical Indication

All four PSMA-targeting PET radiotracers are used to detect PSMA-positive lesions in adult PCa patients. Their clinical indications [42,43,44,46,48,49,50,55,57] are listed as follows

(i) Primary staging of patients with high-risk PCa before curative initial treatment
(ii) The detection of suspected PCa recurrence in patients with rising PSA levels after a curative initial treatment (e.g., radical prostatectomy–RP, external beam radiation therapy–EBRT).

In addition, [^68^Ga]Ga-PSMA-11 has the regulatory indication for selecting patients with metastatic castration-resistant PCa (mCRPC) for potential targeted radionuclide therapy with [^177^Lu]Lu-PSMA-617 (Pluvicto^®^, Novartis AG, Basel, Switzerland).

The primary staging of high-risk PCa patients is conducted with PSA levels of ≥20 ng/mL, a Gleason score ≥ 8 and a clinical stage of T3/T4 (extraprostatic extension/seminal vesicle invasion). Despite early intervention, 20–50% of PCa patients will experience biochemical recurrence within ten years after curative treatment [58,59]. For recurrence monitoring, PSA levels are tested regularly and are used to diagnose the biochemical recurrence (BCR) of PCa. Most guideline associations and medical societies agree on base PSA thresholds as a first indication to BCR, which are defined as PSA levels ≥0.2 ng/mL with a confirmed rise (post-RP) and as a PSA increase of ≥2 ng/mL over PSA nadir (post-EBRT) in accordance with the Phoenix criteria [60,61,62,63,64,65]. While PSA levels are crucial in assessing recurrence, additional factors such as Gleason score, pathological stage, surgical margin and lymph node status also need to be taken into consideration [66,67].

### 3.2. Application

All four described radiotracers share common clinical regulations based on their prescription sheets [42,43,44,46,48,49,50,55,57]. As a safety standard, personnel, trained in radionuclide handling, must ensure drug quality, sterile techniques, radiation shielding, and waste management. If any particles or discolorations to the imaging samples are present, they are not to be used.

The administration is performed intravenously (IV) on PCa patients. To ensure a full dose delivery of [^18^F]DCFPyL, [^18^F]PSMA-1007, and [^18^F]rhPSMA-7.3, a 0.9% sterile saline solution might be used to either dilute the agent sample or flush the IV line post-injection (p.i.). The dilution volume of [^68^Ga]Ga-PSMA-11 is dependent on the applied generator used for the sterile cold kit preparation. Patients must hydrate well, void frequently p.i. and prior to image acquisition to reduce radiation exposure and imaging artifacts. To improve renal clearance, diuretics may be administered simultaneously to reduce unwanted ureter and bladder activity.

The image acquisition is conducted with patients in supine position with arms above the head and with standardized scanning ranges from the mid-thigh area to the vertex of the skull. Besides their radioisotopes, the different imaging agents have key differences in terms of dose preparation, injection volumes, and p.i. imaging windows. An overview of these parameters for each imaging agent is shown in Table 3.

### 3.3. Pharmacology, Pharmacokinetics and Toxicology

All discussed compounds are synthetic peptidomimetics, featuring a urea-based core moiety (Glu-HN-CO-NH-Lys/Glu) binding with high affinity at PSMA epitopes. The overall pharmacokinetics of these compounds can be additionally influenced through the choice of linkers and radiolabeling sites [68]. The tracers bind to PSMA, significantly overexpressed on most PCa cells (e.g., mCRPC) [69] and are being internalized rapidly [70], enabling a strong uptake to metastases. Since a correlation between PSMA and cancer stage and grade has been revealed, showing enhanced PSMA levels with advanced cancer [71,72,73,74,75], a more efficient diagnosis, or even therapy, using PSMA-based agents might be enabled. However, PSMA is also present on healthy tissue and organs, even though lowly expressed. Still, this might lead to non-specific uptakes giving false positives or making distinct imaging challenging. In difficult cases, using additional CT scans to complement the PET images is advised.

All compounds distribute rapidly via the blood stream and show significant uptake to PCa tumor lesions. Despite these shared traits, the pharmacokinetic profiles of these PET tracers vary significantly. This is most evident in the excretion pathways, which is due to compound properties; e.g., [^18^F]rhPSMA-7.3, [^68^Ga]Ga-PSMA-11, and [^18^F]DCFPyL are renally excreted, while [^18^F]PSMA-1007 is primarily hepatobiliary excreted [76,77]. As a result, the latter shows less bladder activity, improving imaging results in the pelvic area due to favorable tumor-to-background ratios [78].

However, these structural differences also impact binding kinetics. In general, all aforementioned PSMA-PET agents exhibit irreversible binding kinetics to tumor lesions. Nevertheless, studies revealed that [^18^F]PSMA-1007 not only binds irreversibly to tumor lesions, but also to organs and tissues [79], resulting in higher mean absorbed doses of non-target tissue. Based on general radiation safety measures, the mean absorbed doses to healthy organs and tissues from PSMA-PET imaging agents are of main concern and are listed in Table 4.

These PSMA-PET imaging agents have no toxicologic safety concerns, as verified in previous animal and clinical studies [42,43,44,46,48,49,50,55,57]. Administered PET tracer concentrations are too low and not intended for continuous use. Hence, doses remain a singular instance with no long-term health effects expected. In less than 1% of patient cases, minor adverse effects, e.g., nausea, diarrhea, dizziness, were observed.

Taking the effective doses of all PSMA-PET imaging agents into consideration, their mean effective doses are in ranges of 0.0121–0.191 mSv/MBq, resulting in effective doses in ranges of 4.1–5.3 mSv per injection, clearly within safety margins of diagnostic routines. Organs absorbing the highest doses oftentimes include the kidneys and the liver (Table 5), with the aim of using the smallest quantity necessary to get the desired information. The applied PSMA-PET imaging agents are designed to deliver the lowest amount of ionizing radiation possible, causing the least risk of cancer and other abnormalities. Overall, delivered radiation doses, at diagnostic levels, are manageable and not a significant safety concern to PCa patients.

### 3.4. Comparative Studies

**Figure 2:**
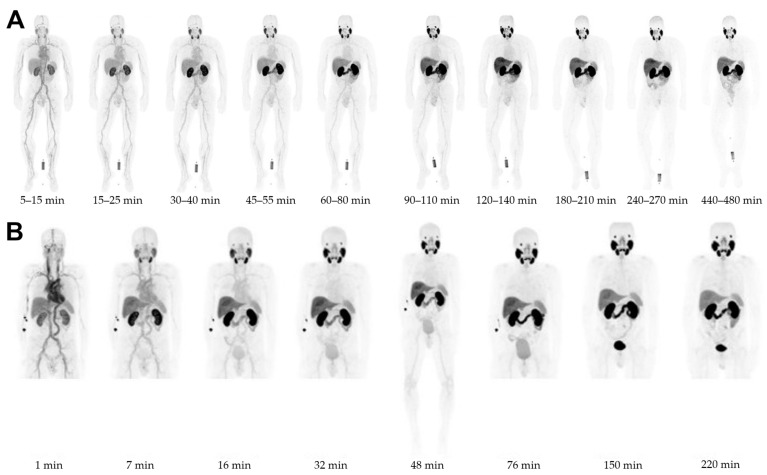
Biodistribution in healthy volunteers utilizing (**A**): [^18^F]PSMA-1007 PET (220 MBq). Originally published in *Eur. J. Nucl. Med. Mol. Imaging* © Springer Nature [78]. (**B**): [^18^F]rhPSMA-7.3 PET (220 MBq). Originally published in *J. Nucl. Med*. © SNMMI [80].

Regarding the biodistribution of the described PSMA-PET radiotracers in healthy volunteers, [^68^Ga]Ga-PSMA-11, [^18^F]rhPSMA-7.3, and [^18^F]DCFPyL presented high uptake in the kidneys and salivary glands, as well as moderate uptake in the liver, bladder, spleen and duodenum. This observation is in correlation with the natural known expression of PSMA in these healthy tissues. These three radiotracers present a similar biodistribution profile which is quite different from the one of [^18^F]PSMA-1007. [^18^F]PSMA-1007 presents high uptake in the kidneys, salivary glands, liver and duodenum, and moderate uptake in the bladder and the spleen. A fast renal clearance for [^68^Ga]Ga-PSMA-11, [^18^F]rhPSMA-7.3, and [^18^F]DCFPyL was also observed. [^18^F]rhPSMA-7.3 has a lower average urinary excretion than [^18^F]DCFPyL and [^68^Ga]Ga-PSMA-11, which can improve imaging in the pelvic region [80]. Penny et al. confirmed this observation, as they measured a lower bladder uptake compared to [^18^F]DCFPyL and [^68^Ga]Ga-PSMA-11 [81]. Nevertheless, the rapid excretion in the urine can also be seen as a drawback, as it causes substantial accumulation in the urinary bladder which can prevent a good visualization of lesions in the pelvic region [82]. In this case, a diuretic agent can be proposed to patients. In comparison, [^18^F]PSMA-1007 presents hepatobiliary clearance which causes a longer residential time and consequently a higher dose in the organs with high uptake. This gives the advantage of having a lower uptake in the pelvic region and allows for a better visualization of small lesions. For all four radiotracers, the uptake in the liver, intestines and kidneys can prevent the visualization of small lesions in these regions. The moderate-to-high uptake in the liver can prevent the detection of liver metastases which indicate a bad prognosis. In comparison with [^18^F]fluorocholine and [^11^C]choline that have high liver uptake, the moderate liver uptake of [^18^F]rhPSMA-7.3 and [^68^Ga]Ga-PSMA-11 allow for a better visualization of liver metastases. A visual comparison of the biodistribution of [^18^F]PSMA-1007 and [^18^F]rhPSMA-7.3 in healthy volunteers is presented Figure 2.

**Figure 3:**
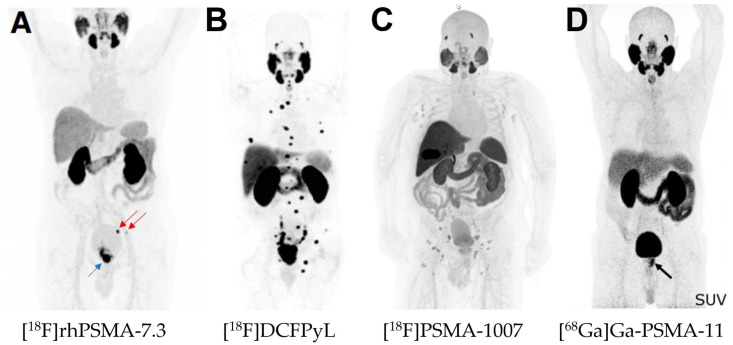
Comparison of PET imaging outcomes in PCa patients using the four approved PSMA-targeting PET radiotracers. (**A**): A 72-y-old patient with high-risk PCa (iPSA, 44 ng/mL) who underwent [18F]rhPSMA-7.3 PET/CT (335 MBq). Images showed primary tumor (blue arrow) and pelvic metastases (red arrows) histologically confirmed after radical prostatectomy. Originally published in *J. Nucl. Med.*, © SNMMI [83]. (**B**): A patient with high risk PCa (iPSA, 13.68 ng/mL, and biopsy Gleason score, 4D5) who underwent [18F]DCFPyL PET/CT (333 MBq). Images showed multifocal osseous lesions involving spine, ribs, pelvis and right clavicle. On subsequent biopsy of transverse process of L3, osseous metastatic (M1b) disease was confirmed. Originally published in *J. Urol.* © American Urological Association [84]. (**C**): A patient with metastatic PCa (Gleason Score 7a, PSA 12 ng/mL) who underwent [^18^F]PSMA-1007 PET/CT (217 MBq, 90 min p.i.). Image showed PSMA avid multifocal primary tumor and several moderate to intensive PSMA avid lymph nodes, no bone or distant metastases detected [85]. (**D**): A patient referred for primary staging of PCa who underwent [68Ga]Ga-PSMA-11 PET/CT (2 MBq/kg). Image showed primary disease (black arrow). Originally published in *EJNMMI Res.* © SpringerOpen [79].

Clinical trials of the four PSMA-PET agents are summarized in Table 6 which presents the major observations during these trials. These four radiotracers have successfully shown their safety at injected doses between 2–4 MBq/kg and their abilities to detect primary PCa lesions and metastases. Examples of successful detection of PCa lesions are presented in Figure 3. It is important to note that each study has been made in different centers with different activities applied and different machines and protocols of imaging used.

At high PSA level (>5 ng/mL), Afshar-Oromieh et al. demonstrate a detection rate of 90% for [^68^Ga]Ga-PSMA-11 in a study involving 319 PCa patients with similar rates for the three other PSMA-PET tracers [86]. For the four PSMA-PET agents, a correlation between PSA level and detection rate was observed: the lower the PSA level, the lower the detection rate. For example, Fendler et al. reported these following values for [^68^Ga]Ga-PSMA-11: 38% for PSA < 0.5 ng/mL (*n* = 136), 57% for 0.5 ≤ PSA < 1.0 ng/mL (*n* = 79), 84% for 1.0 ≤ PSA < 2.0 ng/mL (*n* = 89), 86% for 2.0≤ PSA < 5.0 ng/mL (*n* = 158) and 97% for PSA > 5.0 ng/mL (*n* = 173, *p* < 0.001) [87]. In a study with [^68^Ga]Ga-PSMA-11, Eiber et al. reported different values compared to Fendler et al.: 96.8% (120/124) for PSA < 2 ng/mL, 93.0% (67/72) for 1 < PSA < 2 ng/mL, 72.7% (24/33) for 0.5 < PSA < 1 ng/mL, and 57.9% (11/19) for 0.2 < PSA < 0.5 ng/mL) [88]. This observation can be due to the heterogeneity of the PCa patients. On the other hand, Morris et al. reported these values for [^18^F]DCFPyL: 36.2% for PSA < 0.5 ng/mL to 96.7% for PSA ≥ 5 ng/mL [89]. For [^18^F]PSMA-1007, these values have been reported: 60% for PSA < 0.2 ng/mL, 70–65% for 0.6 ≤ PSA < 1.2 ng/mL and 80% for PSA >1.2 ng/mL [90]. In comparison, [^18^F]rhPSMA-7.3 and [^18^F]PSMA-1007 present higher detection rates (64% and 60%, respectively) compared to [^18^F]DCFPyl (36%) and [^68^Ga]Ga-PSMA-11 (38%) for patients with very low PSA level (<0.5 ng/mL). As one third of PCa patients will experience BCR within 5 years of treatment, being able to detect lesion in BCR patients is of high importance [91].

Unspecific bone uptake (UBU) has been observed in some patients, which complicates the diagnosis of bone metastasis. Rizzo et al. describe the UBU of these four PSMA-PET radiotracers via the use of homunculus [92]. The three main regions of UBU are the ribs (57.5%, 202/348 patients), the pelvis (24.8%, 87/348 patients) and the spine (9.7%, 34/348 patients) [93]. [^18^F]PSMA-1007 presents significatively more UBU than the three other approved agents, especially in the pelvic region. Due to the recurrence of these benign lesion detections using [^18^F]PSMA-1007, patients need to undertake another PET exam using one of the three other radiotracers, notably [^68^Ga]Ga-PSMA-11. In fact, in a retrospective study, Dias et al. have observed no UBU for the patients imaged with [^68^Ga]Ga-PSMA-11 and UBU notably in the ribs for the patients imaged with [^18^F]PSMA-1007 [79]. A retrospective study of dual time point biphasic PET/CT (1 and 3 h p.i.) shows that biphasic images help in the differentiation between malign and benign lesions [94].

Before the approval of PSMA-PET tracers, PET scans were already conducted using the approved radiotracers [^18^F]FDG, [^18^F]fluorocholine and [^11^C]choline to detect PCa lesions. These three radiotracers can be used to diagnose a large panel of cancer types and other pathologies and are not targeting specifically PSMA. Multiple studies have been conducted to compare these gold standards and more general tracers with these four PSMA-PET tracers. For example, PET/CT scans of 37 patients with BCR PCa injected with both [^68^Ga]Ga-PSMA-11 and [^18^F]fluorocholine showed a significant improvement of image contrast with [^68^Ga]Ga-PSMA-11 compared to [^18^F]fluorocholine. This study concluded in significantly higher lesion detection, mostly for metastases, even in small lymph nodes or liver metastases due to lower background uptake in these regions even at low PSA level [95]. Similarly, a retrospective study on 257 BCR patients showed that [^68^Ga]Ga-PSMA-11 outperformed [^18^F]FDG for low PSA levels (≤1 ng/mL). Nevertheless, for higher PSA level, the superiority of [^68^Ga]Ga-PSMA-11 is less evident [96]. Regarding [^18^F]FDG, a meta-analysis of 27 literature studies including 2891 patients concluded that [^68^Ga]Ga-PSMA-11 and [^18^F]PSMA-1007 outperformed [^18^F]FDG for lesion detections [97].

In parallel, [^18^F]fluciclovine, a clinically approved non-PSMA-based PET imaging agent is also used for PCa detection [98]. Comparative studies with PSMA-PET tracers were also performed to evaluate their relative efficiency of detection. Multiple studies concluded a higher performance of the PSMA-PET tracers compared to [^18^F]fluciclovine in the detection of primary tumors, small lesions and metastases even at a low PSA level (<0.5 ng/mL) [99]. These comparative studies confirm one more time the power of PSMA-PET agents in PCa detection. A non-exhaustive list of comparative clinical trials is provided in Table 7.

**Table 6:** Clinical characteristics and outcome of the clinical trials of the four approved PSMA-targeting PET radiotracers (non-exhaustive list).

Imaging Agent	Clinical Trial	Phase	Publication Year	Number of Patients	Type of Patient	Main Findings	Ref.
**Posluma**^®^: [^18^F]rhPSMA-7.3	NCT03995888	1	2021	6	Healthy volunteers	No clinically detectable pharmacological effects were observed after injection of 220 MBq. The mean effective dose received by the patients was 0.0141 mSv/MBq which is favorable for diagnostics by PET and lower than for other PET PSMA agents.	[80]
	LIGHTHOUSE NCT04186819	3	2021	335	High risk PCa	Confirmation of the safety and accuracy of detection of lesions in high risk PCa in patients with a large range of PSA levels. Confirmation of the safety and accuracy of detection of lesions in recurrent PCa in patients with a large range of PSA levels.	[81]
	SPOTLIGHT NCT04186845	3	2021	389	Recurrent PCa		[100] [101]
**Radelumin**^®^: [^18^F]PSMA-1007		1/2	2017	3 10	Healthy volunteers PCa patients	Comparable performance of [^18^F]PSMA-1007 and [^68^Ga]Ga-PSMA-11.	[102]
		1/2	2023	3 13	Healthy volunteers PCa patients	Safe and well tolerated tracer, high accuracy in the diagnoses for primary and metastatic lesions.	[103]
		2	2019	40	After RP or radiation beam therapy	Scan positivity is dependent on PSA level. Moderate detection of tumor lesion (60%) at low PSA levels < 0.2 ng/mL.	[90]
		3	2021	175	PCa	Detection efficiency of 80%.	[104]
**Locametz**^®^/ **Illuccix**^®^/**Gozellix^TM^:** [^68^Ga]Ga-PSMA-11	NCT03001869		2023			Completed–No results posted.	
	NCT02659527	3	2018	145	BCR PCa patients after RP	Detection efficiency of 85%.	[105]
	NCT03353740 and NCT02940262	3	2020	635	BCR PCa patients after RP and/or radiation therapy	High detection rates of 75% and positive prediction values between 84 and 92%.	[87]
		3	2017	1007	Recurrent PCa	No adverse clinical side effects observed. Same conclusions, significantly higher detection of lesions compared to other already approved tracers, detection rate increases as the PSA level increases.	[106]
	NCT03582774 PSMA-SRT	3	2025	193	Recurrent PCa after RP	Actively recruiting for [^68^Ga]Ga-PSMA-11 PET/CT for PCa salvage radiotherapy planning.	[107]
			2015	70	Restaging PCa	Good detection rate even at low PSA level (85%).	[108]
			2015	248	BCR PCa patients after RP	PSA level ≥ 0.2 ng/mL. [^68^Ga]Ga-PSMA-11 surpasses other imaging modalities for restaging PCa detection and higher detection efficacy compared to other tracers. 89.5% of detection when PSA level ≥ 1.0 ng/mL.	[88]
					BCR PCa patients after RP	[^68^Ga]Ga-PSMA-11 surpasses other imaging modalities for restaging PCa detection and higher detection efficacy compared to other tracers as concluded by Eiber et al. [88].	[109]
**Pylarify**^®^/**Pylclari**^®^: [^18^F]DCFPyL	NCT02523924	2	2020	31	RP BCa patients	Prospective study, proved good detection of lesions even at low PSA level.	[110]
		2	2018	248	BCR PCa patients	Early detection even at low PSA level (<0.5 ng/mL). Results comparable to those of [^68^Ga]Ga-PSMA-11 and [^18^F]PSMA-1007.	[111]
	NCT03181867	2	2019	800	BCR patients	90% detection rate for PSA level > 0.5 ng/mL.	[112]
	NCT02899312	3	2019	2244	BCR after RP	This study confirmed the safety of use and the good sensitivity of this tracer for small lesion detection	[113]
	OSPREY NCT02981368	2/3	2021	385	high risk and recurrent/metastatic PCa.	High positive prediction values (78.1–90.5%).	[84]
	CONDOR NCT03739684	3	2021	208	BCR PCa after RP of radiotherapy	Confirmation of the accuracy of recurrence or metastases lesion detection from previous clinical trials, study even with patients with low PSA levels. [^18^F]DCFPyL has similar safety and performance compared to [^68^Ga]Ga-PSMA-11.	[89]
	NCT03232164	3	2024	167		Pilot studies for PET/CT and PET/MRI. No results posted.	

**Table 7:** Comparative clinical studies between approved PSMA- and non-PSMA-based PET radiotracers (non-exhaustive list).

Imaging Agent	Clinical Trials	Phase	Year	Number of Patients	Type of Patient	Main Findings	Ref.
**[^68^Ga]Ga-PSMA-11** and **[^18^F]fluorocholine**			2016	32	BCR PCa patient after RP	43% of patients with a negative [^18^F]fluorocholine PET/CT scan have been diagnosed with PCa lesions using [^68^Ga]Ga-PSMA-11 PET/CT and confirmed with other diagnosis techniques. [^68^Ga]Ga-PSMA-11 outperformed [^18^F]fluorocholine.	[91]
**[^18^F]PSMA-1007** and **[^18^F]fluorocholine**	NCT04102553	3	2022	190	BCR PCa patients	Significantly higher detection rate for [^18^F]PSMA-1007 than [^18^F]fluorocholine especially at low PSA levels.	[114]
	NCT04742361	3	2024			Actively recruiting.	
**[^18^F]PSMA-1007** and **[^18^F]FDG**			2024	42	RP PCa	[^18^F]PSMA-1007 outperformed the detection of primary tumors in the prostate glands.	[115]
			2021	21		Significantly more tumor lesion and benign lesion uptake for [^18^F]PSMA-1007.	[116]
**[^68^Ga]Ga-PSMA-11** and **[^18^F]fluciclovine**	NCT03515577		2020	50	BCR PCa	Detection rate in patients with low PSA level (≤2.0 ng/mL) is significantly lower for [^18^F]fluciclovine than [^68^Ga]Ga-PSMA-11 and up to twice lower for lesions in the pelvic lymph nodes region.	[117]
**[^18^F]PSMA-1007** and [**^18^F]fluciclovine**	NCT04239742	2	2020	50	PCa	[^18^F]PSMA-1007 outperformed [^18^F]fluciclovine to detect lesions (68% vs 42%).	[118]
**[^18^F]PSMA-1007** and **[^68^Ga]Ga-PSMA-11**	NCT05079828	3	2024	100	BCR PCa patient after RP	No results posted.	[119]
**[^18^F]PSMA-1007** and **[^18^F]DCFPyL**			2018	12	Drug-naive or before surgery PCa patients	Equivalence of efficacy for both tracers. [^18^F]PSMA-1007 clearance pathway is an advantage for pelvic metastases detection.	[120]

## 4. Further Candidates and Outlook

Several other PSMA-PET ligands have been developed and undertaken for clinical evaluation. [^18^F]DCFBC is a ^18^F-PSMA targeting agent based on the recognition pattern Glu-NH-O-NH-Ser which has been evaluated in clinical trials before its second generation [^18^F]DCFPyL [121,122]. This latter has outperformed [^18^F]DCFBC, notably with a lower background uptake, more tumor uptake and better correlation between uptake and the Gleason score. Due to the significantly better performance of [^18^F]DCFPyL, no further clinical studies were conducted. Neumaier and coworkers have developed [^18^F]JK-PSMA-7 derived from [^18^F]DCFPyL. It has a highly similar biodistribution pattern compared to [^18^F]DCFPyL and [^68^Ga]Ga-PSMA-11, but PCa patient phase I studies have highlighted the higher edge-contrast, resolution, and signal-to-noise ratio of [^18^F]DFCPyL notably for small lesions [123,124]. Piron et al. have successfully assessed [^18^F]PSMA-11 during phase II clinical trials (NCT03573011) [125]. Its safety usage was confirmed (injection of 2–4 MBq/kg) as well as the detection of PCa lesions. Nevertheless, a diuretic agent was used to evaluate lesions in the proximity of ureters. The NOTA equivalent of PSMA-617, called PSMA-BCH, has been radiolabelled with ^18^F-Al and clinically assessed in 11 PCa patients [126]. Renal clearance was observed and a good detection of small lesions showed a good suitability of this ^18^F-tracer for PCa lesion detection. Nonetheless, for now, no ^18^F-PSMA-PET agents have outperformed the three clinically approved tracers.

[^124^I]MIP-1095 has been assessed in PET/CT on 16 mCRPC patients in a theranostic study in combination with [^131^I]MIP-1095. SPECT and PET/CT have been recorded after injection and to monitor the treatment, respectively. Good safety of the PET tracer has been confirmed for an injected dose of 67.4 MBq as no side effects have been observed. High uptake in the kidney and salivary glands and moderate uptake in the liver, intestines and bladder have been observed as well as a renal clearance. This biodistribution is similar to the one of [^68^Ga]Ga-PSMA-11 and clear visualization of the metastases has been demonstrated. The monitoring of treatment has also been successful with the measurement of the reduction in number and size of the metastases [127].

[^68^Ga]Ga-NGUL is a NOTA-PSMA-based PET agent that has successfully passed a phase I clinical trial presenting good safety, fast renal clearance and good capacity to visualize metastases and primary tumors in PCa patients. These promising results could lead to phase II/III clinical trials in the future [128].

For the treatment of mCRPC, [^177^Lu]Lu-PSMA-617 and [^177^Lu]Lu-PSMA-I&T are currently used in clinical routine as therapeutic agents in combination with [^68^Ga]Ga-PSMA-11 as diagnostic agent. Multiple clinical trials have been conducted with [^68^Ga]Ga-PSMA-617 [129,130,131] and [^68^Ga]Ga-PSMA-I&T to develop theranostic pairs with identical ligand structure in order to develop pairs with more similar biodistribution and pharmacological profiles [132,133,134,135]. They showed minor deviations in the biodistribution (lower liver uptake, higher bone and background uptake for [^68^Ga]Ga-PSMA-617 and [^68^Ga]Ga-PSMA-I&T compared to [^68^Ga]Ga-PSMA-11) and no significant difference in tumor uptake [136]. This comparative study confirmed the good suitability of these two tracers to be used in clinical routine for theranostic applications.

Privé et al. have conducted a first-in-human study with [^89^Zr]Zr-PSMA-617 and [^89^Zr]Zr-PSMA-I&T in a BCR PCa patient. Biodistributions were observed to be really similar to their ^177^Lu-analog and cancerous lesions were correctly identified. Thanks to the promising diagnostic capacity of these two ^89^Zr-PSMA tracers, a clinical study is being planned [137].

Similarly, [^64^Cu]Cu-PSMA-617 and [^64^Cu]Cu-PSMA-I&T have been clinically evaluated in the PET detection of lesions in PCa patients. Phase I/II clinical trials showed good safety and excellent capacity to detect PCa lesions for both of these tracers with similar precision in the detection [138,139,140]. A phase III trial is currently ongoing to confirm these promising results (Solar-stage: NCT06235151) [141]. With the development of more efficient production routes for both ^64^Cu and ^67^Cu and the development of personalized medicine thanks to radiotheranostics, it is likely that [^67^Cu]Cu-PSMA-617 and [^67^Cu]Cu-PSMA-I&T will undergo clinical trials in the next decades.

[^61^Cu]Cu-PSMA-I&T and its NODA-GA analog [^61^Cu]Cu-NODA-GA-PSMA-I&T have been preclinically assessed in a PCa rodent model. The NODA-GA analog presented a better biodistribution and excretion profile and higher tumor uptake compared to the DOTA-GA analog. Moreover, the performance of [^61^Cu]Cu-NODA-GA-PSMA-I&T was similar to those of [^68^Ga]Ga-PSMA-11 and [^18^F]PSMA-1007 with the advantage of performing PET at later timepoints. A first-in-human study has been further conducted and has shown good safety of the tracer as well as its ability to detect metastases in mCRPC patients [142]. Considering these promising results, a phase I study has been announced [143].

Yang et al. have assessed [^64^Cu]Cu-PSMA-BCH similarly to their previous [^18^F]F-Al-PSMA-BCH study. Both tracers showed similar biodistribution and a renal excretion pathway with a good capacity in the detection of PCa lesions. Compared to its ^18^F-analog, [^64^Cu]Cu-PSMA-BCH allowed PET at later timepoints (24 h p.i.) after renal excretion which led to better visualization of pelvic lesions [144].

Zhang and coworkers developed a series of PSMA-617-derived tracers for ^64^Cu-PET. The naphthyl moiety of PSMA-617 has been replaced by a quinoline and they have proposed a NOTA version [145,146]. A study with 29 PCa patients has been conducted to compare [^64^Cu]Cu-DOTA-PSMA-3Q and [^64^Cu]Cu-NOTA-PSMA-3Q. Significantly higher salivary glands uptake and lower liver uptake have been observed for the NOTA tracer. This comparison highlights the importance of the choice of the chelating agent toward the biodistribution properties of the tracer which are of high importance for PET or targeted radionuclide therapy applications. The group is currently working on a ^18^F-equivalent of this NOTA tracer.

[^64^Cu]Cu-sar-bisPSMA has been used in clinical trials (Cobra: NCT05249127), notably in a phase II trial in comparison with [^68^Ga]Ga-PSMA-11 (Propeller: NCT04839367) [147,148]. This study demonstrated its good safety and a better efficiency of the ^64^Cu tracer compared to [^68^Ga]Ga-PSMA-11. This is notably due to the higher tumor uptake that leads to the better detection of smaller lesions. The tracer is currently under phase III trials to confirm its efficiency in a larger cohort (clarify: NCT06056830) [148]. The therapeutic analog [^67^Cu]Cu-sar-bisPSMA is currently under clinical trials of phase I/IIa (SECuRE: NCT04868604) [149]. In the case of success in these clinical trials, [^64/67^Cu]Cu-sar-bisPSMA could become the first true theranostic pair for PCa.

A first-in-human application of [^152^Tb]Tb-PSMA-617 has been conducted on one PCa patient [150]. The use of [^152^Tb]Tb-PSMA-617 has been determined as safe and successful in the detection of cancer lesions as [^68^Ga]Ga-PSMA-11, yet with lower image quality. Moreover, [^152^Tb]Tb-PSMA-617 has a similar biodistribution profile compared to [^177^Lu]Lu-PSMA-617, already approved for clinical treatment. The potential use of the terbium sisters in theranostics is huge thanks to their physical properties adapted for clinical purposes and high versatility (^152^Tb: PET, ^155^Tb: SPECT, ^149^Tb: targeted alpha therapy, and ^161^Tb: β^–^- targeted radionuclide therapy). There is a great chance that the first clinical approval of these four terbium sisters will be conducted using a PSMA- or TATE-based ligand.

In the same manner, a first-in-human study has been conducted with [^44^Sc]Sc-PSMA-617 and has shown a similar biodistribution and the same capacity in lesion detection as [^68^Ga]Ga-PSMA-11 [151,152]. Due to the longer half-life of ^44^Sc (4.3 h), this radioligand could be used in image-guided radiosurgery using positron probes intraoperatively. The perspective of this work is to assess mCRPC targeted radionuclide therapy using [^47^Sc]Sc-PSMA-617 to see the potential clinical application of the matched pair ^44/47^Sc in PCa treatment.

Another strategy to increase the diagnostic efficiency for PCa patients and their differentiation is to conduct imaging with a PSMA-PET agent and [^18^F]FDG. Kepenek et al. reported a case study with 93 patients with high risk CRPC, where significantly higher tumor uptake and lesion detection were observed with [^18^F]FDG compared to [^68^Ga]Ga-PSMA-11 [153]. Within these PSMA-/FDG+ patients with high Gleason scores (≥8) and PSA levels (≥7.9 ng/mL), the glucose metabolism of tumors is increased significantly which allows a better diagnosis for this radiotracer combination and better choice of the following treatment, especially for [^177^Lu]Lu- or [^225^Ac]Ac-PSMA-617, that have a poor prognosis in PSMA-/FDG+ patients. This strategy exemplifies so called personalized medicine and is also used to indicate the most adapted treatment for the patients. These observations have been confirmed by the retrospective analysis studies of Khreish et al., Chen et al. and Pan et al. on 29, 56 and 298 CRPC patients, respectively, that underwent both [^18^F]FDG and [^68^Ga]Ga-PSMA-11 PET/CT [154,155,156].

Also, other studies have investigated the dual-tracer approach for PCa diagnostics, notably with [^68^Ga]Ga-PSMA-11 and [^68^Ga]Ga-DOTA-RM2. A case study on 22 BCR PCa patients proved their complementarity and synergy for the detection of small lesions which reflect the heterogeneity of PCa during the staging phase [157].

## 5. Conclusions

This review highlights the significant impact of approved PSMA-PET imaging agents [^68^Ga]Ga-PSMA-11 (Locametz^®^/Illuccix^®^/Gozellix^TM^), [^18^F]DCFPyL (Pylarify^®^/Pylclari^®^), [^18^F]PSMA-1007 (Radelumin^®^), and [^18^F]rhPSMA-7.3 (Posluma^®^) on the landscape of PCa detection and management. These agents have revolutionized clinical practice by offering enhanced sensitivity and accuracy in detecting PCa, leading to improved staging, treatment planning and patient outcomes.

Each radiotracer possesses unique chemical and pharmaceutical characteristics, influencing its application and utility in specific clinical scenarios. [^68^Ga]Ga-PSMA-11, with its established role and wide availability, remains a cornerstone while the ^18^F-labeled agents [^18^F]DCFPyL, [^18^F]PSMA-1007 and [^18^F]rhPSMA-7.3 offer advantages in terms of imaging properties and potential for radiotheranostic applications.

The approval and increasing adoption of these PSMA-targeted PET imaging agents mark a pivotal advancement in personalized medicine for PCa. As clinical experience expands and new data emerge, their role in guiding treatment decisions and improving patient care will continue to evolve, promising a future of more precise and effective interventions in the fight against PCa.

## Figures and Tables

**Table 1:** Names and companies of approved PSMA-targeting PET radiotracers.

Working Name	Trade Name	Other Name	Company
**[^68^Ga]Ga-PSMA-11**	Locametz^®^/Illuccix^®^/Gozellix^TM^	Gallium Ga 68 gozetotide	Novartis AG (Basel, Switzerland)/Telix Pharmaceuticals, Ltd. (Melbourne, Australia)
**[^18^F]DCFPyL**	Pylarify^®^/Pylclari^®^	Piflufolastat F 18	Progenics Pharmaceuticals, Inc. (New York, NY, USA)/Curium PET France SA (Paris, France)
**[^18^F]PSMA-1007**	Radelumin^®^	-	ABX GmbH (Radeberg, Germany)
**[^18^F]rhPSMA-7.3**	Posluma^®^	Flotufolastat F 18 gallium	Blue Earths Diagnostics, Ltd. (Oxford, UK)

**Table 2:** Release specifications of approved ^18^F-labeled PSMA-targeting PET radiotracers.

Imaging Agent	pH Value	Radioactivity Concentration [MBq/mL]	Amount of Carrier [µg/GBq]
**Radelumin**^®^: [^18^F]PSMA-1007	4.5–8.5	1300 or 2000	-
**Pylarify**^®^/**Pylclari**^®^: [^18^F]DCFPyL	4.5–7.5 (EU) 4.5–7.0 (US)	1000 or 1500 (EU) 37–2960 (US)	- 0.27 (US)
**Posluma**^®^: [^18^F]rhPSMA-7.3	4.0–6.0	296–5846	3.42

**Table 3:** Comparison of application parameters of the described PSMA-targeting PET radiotracers.

Imaging Agent	Recommended Activity for Adults per Injection	Volume Limit [mL]	Imaging Window [min p.i.]
**Radelumin**^®^: [^18^F]PSMA-1007	**EMA**: 3.6–4.4 MBq/kg e.g., 70 kg patient: 252–308 MBq Maximum: 450 MBq regardless of patient weight	≤10	90–120
	**FDA**: Not approved yet		
**Posluma**^®^: [^18^F]rhPSMA-7.3	**EMA**: Not approved yet	≤5 (undiluted)	60
	**FDA**: Fixed dose of 296 MBq No weight-based adjustments		
**Locametz**^®^/**Illuccix**^®^/**Gozellix^TM^:** [^68^Ga]Ga-PSMA-11	1.8–2.2 MBq/kg Minimum: 111 MBq Maximum: 259 MBq	None ^†^	50–100
**Pylarify**^®^/**Pylclari**^®^: [^18^F]DCFPyL	**EMA**: 3–5 MBq/kg e.g., 70 kg patient: 210–350 MBq Minimum: 190 MBq Maximum: 360 MBq	0.2–10	90–120
	**FDA**: Recommended dose of 333 MBq Minimum: 296 MBq Maximum: 370 MBq	None ^†^	60–90

^†^ Volume is calculated based on calibration time, required dose and/or utilized generator, e.g., for Locametz^®^ kit preparation.

**Table 4:** Estimated radiation absorbed doses in organs/tissues in adult PCa patients who received one of the four PSMA-targeting PET radiotracers.

Critical Organ	Radelumin^®^ [^18^F]PSMA-1007	Posluma^®^ [^18^F]rhPSMA-7.3	Locametz^®^/Illuccix^®^/Gozellix^TM^ [^68^Ga]Ga-PSMA-11	Pylclari^®^/Pylarify^®^ [^18^F]DCFPyL ^†^
	Mean Absorbed Doses [mGy/MBq]			
**Adrenals**	0.0349	0.184	0.0156	0.0326
**Bone surfaces**	N.A.	N.A.	N.A.	0.00662
**Brain**	0.0030	0.002	0.0104	0.00215
**Breast**	N.A.	0.004	0.0103	0.00767
**Gallbladder**	0.1400	0.017	0.0157	0.0255
**Gastrointestinal tract**				
**Stomach**	0.0170	0.012	0.0129	0.0127
**Small intestine**	0.0334	0.012	0.014	0.0101
**Lower colon**	0.0239	0.007	0.0134	0.0101
**Upper colon**	0.0176	0.01	N.A.	0.0125
**Heart wall**	0.0259	0.02	0.012	0.0178
**Kidneys**	0.1030	0.0172	0.3714	0.124
**Liver**	0.0883	0.062	0.0409	0.0388
**Lungs**	0.0147	0.01	0.0111	0.0121
**Muscles**	0.0103	0.006	0.0103	0.00714
**Pancreas**	0.0677	0.028	0.0147	0.0183
**Red marrow**	0.0121	0.01	0.0114	0.00851
**Skin**	N.A.	0.002	0.0091	0.0054
**Spleen**	0.0851	0.083	0.065	0.0283
**Testes**	0.0074	0.005	0.0111	0.00638
**Thymus**	0.0106	0.01	0.0105	0.00769
**Thyroid**	0.0151	0.01	0.0104	0.00687
**Urinary bladder**	0.0212	0.006	0.0982	0.00712
**Effective dose [mSv**/**MBq]**	0.0191	0.014	0.0169	0.0121

^†^ Mean absorbed doses shown for Pylclari^®^ based on most recent prescribing information [57].

**Table 5:** Effective and critical radiation doses of each PSMA-targeting PET radiotracer.

Imaging Agent	Administered Activity [MBq]	Effective Dose [mSv]	Critical Organs	Radiation Doses [mGy]
**Radelumin**^®^: [^18^F]PSMA-1007	280 ^A^	5.3	Gallbladder	39
			Kidneys	29
			Liver	25
**Posluma**^®^: **[**^18^F]rhPSMA-7.3	296 ^B^	4.1	Adrenal glands	54.3
			Kidneys	51
			Submandibular glands	43.8
**Locametz**^®^/**Illuccix**^®^/**Gozellix**^TM^: [^68^Ga]Ga-PSMA-11	259 ^C^	4.4	Kidneys	96.2
			Urinary bladder	25.4
			Spleen	16.8
**Pylarify**^®^/**Pylclari**^®^: [^18^F]DCFPyL	360 ^D^	4.4	Kidneys	44.6
			Liver	14
			Spleen	10.2

^A^ Recommended activity for an adult weighing 70 kg, with 280 MBq representing the mean of 252–308 MBq; ^B^ fixed dose; ^C^ max. recommended overall activity regardless of body weight; ^D^ max. recommended activity for an adult weighing 70 kg (EMA).

## Data Availability

Not Applicable.
